# Reduced miR-146a Promotes REG3A Expression and Macrophage Migration in Polymyositis and Dermatomyositis

**DOI:** 10.3389/fimmu.2020.00037

**Published:** 2020-02-21

**Authors:** Tingwang Jiang, Yuanlan Huang, Haohao Liu, Qiangwei Xu, Yanping Gong, Yao Chen, Xiaowei Hu, Zhijun Han, Mingzhu Gao

**Affiliations:** ^1^Key Laboratory, The Second People's Hospital of Changshu, Changshu, China; ^2^Department of Clinical Immunology, Institution for Laboratory Medicine, Changshu, China; ^3^Department of Laboratory Medicine, No. 455 Hospital of the Chinese People's Liberation Army, Shanghai, China; ^4^Department of Laboratory Medicine, The Affiliated Wuxi No.2 People's Hospital of Nanjing Medical University, Wuxi, China; ^5^Department of Rheumatology, The Affiliated Wuxi No.2 People's Hospital of Nanjing Medical University, Wuxi, China; ^6^Affiliated Wuxi Clinical College of Nantong University, Wuxi, China

**Keywords:** miR-146a, REG3A, macrophage migration, polymyositis and dermatomyositis, autoimmune disease

## Abstract

**Background:** Growing evidence from studies elsewhere have illustrated that microRNAs (miRNAs) play important roles in polymyositis and dermatomyositis (PM/DM). However, little has been reported on their relationship with regenerating islet-derived protein 3-alpha (REG3A) as well as their associative roles in macrophage migration. Therefore, this study sought to establish the association between miR-146a and REG3A as well as investigate their functional roles in macrophage migration and PM/DM pathogenesis.

**Methods:** Peripheral blood mononuclear cells (PBMCs) were isolated from PM/DM patients and healthy controls through density centrifugation. Macrophages were obtained from monocytes purified from PBMCs via differentiation before their transfection with miRNA or plasmids to investigate cell migration with transwell assay. An experimental autoimmune myositis murine model was used to investigate PM/DM. Real-time PCR and Western blot analysis were conducted to determine the expression levels of miR-146a, interferon gamma (IFN-γ), interleukin (IL)-17A, and REG3A.

**Results:** The messenger RNA (mRNA) expression level of miR-146a markedly decreased, while the mRNA level of REG3A, IFN-γ, and IL-17A expression increased substantially in PBMCs from PM/DM patients compared with the healthy controls. The levels of IFN-γ and IL-17A in serum from PM/DM patients was much higher than the healthy controls. Immunohistochemistry analysis showed that REG3A expression increased in muscle tissues from patients. Consistent with clinical data, the mRNA expression level of miR-146a also decreased, whereas the mRNA and protein level of REG3A, IFN-γ, and IL-17A significantly increased in the muscle tissues of experimental autoimmune myositis mice. Moreover, miR-146a inhibited monocyte-derived macrophage migration, and REG3A promoted macrophage migration. In addition, IL-17A induced REG3A expression, while miR146a inhibited expression of REG3A in monocyte-derived macrophages from the PBMCs of the healthy donors. Notably, inhibition of macrophage migration by miR-146a was via the reduction in REG3A expression.

**Conclusions:** Reduced miR-146a expression in PM/DM leads to increased REG3A expression that increases inflammatory macrophage migration, which may be a possible underlying mechanism of DM/PM pathogenesis.

## Highlights

- miR-146a is decreased and REG3A is increased in PM/DM patients.
- REG3A promotes macrophage migration, while miR-146a inhibits migratory capacity of macrophage.
- miR-146a inhibits macrophage migration via reduction in REG3A expression.

## Introduction

Polymyositis and dermatomyositis (PM/DM), diseases of the connective tissues that are classified as typical idiopathic inflammatory myopathies, affect mainly proximal muscles and other body parts such as skin, lungs, esophagus, heart, and joints (1, 2). Although the clinical differentiation of DM from PM is difficult, the former discern from the latter based on classical cutaneous manifestations such as heliotrope rash, shawl sign, as well as Gottron's papules and signs (3). Usually, PM/DM are considered as separate autoimmune diseases, albeit some overlap with other disorders such as rheumatoid arthritis, systemic sclerosis, and systemic lupus erythematosus (1). To support this assertion, a previous report suggested the role of persistent monocytes/macrophages markers in these disease processes (4). Besides, DM is the most common form of classical inflammatory myopathy, while isolated PM is rare but frequent misdiagnosis (5). Therefore, detailed understanding of the exact pathological processes of PM/DM could be valuable for determining appropriate treatment options for patients suffering from the aforementioned conditions who are at 120% increased mortality risk (6, 7).

The pathogenic roles of microRNAs have been extensively investigated in malignant diseases as well as in autoimmune disorders. Although many microRNAs levels altered in PM/DM patients after treatment, little study reported the role of microRNA in PM/DM (8). So far, miR-21, miR-381, and miR-146a have been reported to regulate macrophage migration in PM/DM (9–11). However, the regulatory mechanism of these microRNAs in PM/DM remains unclear. miR-146a, a member of the microRNA precursors (family miR-146) found in mammals, is located on chromosome 5, which has been observed to play critical anti-inflammatory role by modulating nuclear factor kappa-light-chain-enhancer of activated B cells signal pathway activation as well as release of toll-like receptor 4-induced inflammatory factor (12). The modulation of the aforesaid physiological processes by miR-146a culminated in further downregulation of downstream inflammatory mediators such as interleukins (IL-1β, IL-6, and IL-8) coupled with tumor necrosis factor alpha, thereby preventing overreaction induced by negative immuno-inflammatory regulation (13–15). In this regard, Yin et al. reported the regulation of inflammatory macrophage infiltration in PM/DM by miR-146a via tumor necrosis factor receptor-associated factor 6 targeting and the IL-17/intercellular adhesion molecule 1 pathway dysregulation (11). It is described earlier that deficiency of miR-146a could result in increased expression of macrophages (M1) activation biomarkers with concomitant upregulation of proinflammatory cytokines in diabetic miR-146a^−/−^ mice (16). Likewise, Peng et al. had previously established the involvement of muscle macrophage infiltration in PM/DM pathogenesis (17). In a related study, macrophage polarization in systemic juvenile idiopathic arthritis by miR-146a via the M1-associated gene, inhibin beta A subunit targeting (18).

Regenerating islet-derived protein 3-alpha (REG3A), also known as heptocarcinoma–intestine–pancreas or pancreatic-associated protein is a serum biomarker released from the gut upon injury to function as an antimicrobial protein by resisting bacterial proliferation at the injured site (19, 20). Beside the antimicrobial function, RegIIIγ expression, a mouse homolog of human REG3A, has been established to increase after a mucosal damage and hepatic injury with potential effect on tissue regeneration (21, 22). Likewise, it has been revealed that REG3A expression by skin keratinocyte was induced by IL-17 with possible mediation in epidermal hyperproliferation in psoriatic wound repair (23). Meanwhile, inhibition of REG3A expression in diabetic mice exacerbated toll-like receptor 3-mediated skin inflammation (21, 24). However, its role in inflammatory macrophage infiltration in PM/DM has not been elucidated. In this regard, the actual mechanistic role of miR-146a and its relationship with REG3A in the regulation of inflammatory macrophage migration to weakened muscle tissues in PM/DM pathophysiology awaits exploration.

Herein, this study aimed to investigate the function of miR-146a and its association with the level of REG3A in regulating macrophage migration in PM/DM patients vis-à-vis the healthy controls. The present study also sought to explore the role of IL-17A in the induction of REG3A expression.

## Materials and Methods

### Recruitment of PM/DM Patients and the Healthy Controls

This study involved subjects (PM/DM patients and the healthy controls) recruited from inpatients at the Department of Laboratory Medicine, The Affiliated Wuxi NO.2 People's Hospital of Nanjing Medical University (Wuxi, Jiangsu Province, China) between 2016 and 2017. Peripheral blood was obtained from 20 healthy controls and 25 patients with PM/DM (6 PM, 19 DM; 8 male, 17 female; mean age, 37 ± 13 years). The PM/DM patients were age and gender matched with the healthy controls. The PM/DM patients were diagnosed via determination of their serum samples and muscle biopsy results according to the European Neuromuscular Center pathology diagnosis criteria as described elsewhere (25). The experimental protocol was approved by the Ethics Committee of The Affiliated Wuxi No. 2 People's Hospital of Nanjing Medical University. All patients provided written informed consent for the use of their tissues and data before the study.

### Experimental Autoimmune Myositis Model

Adult (6–8 weeks old) Balb/c female mice were obtained from Jiangsu Synthgene Biotechnology Co., Ltd and maintained with free access to pellet foods and water under controlled conditions (22°C, 55% humidity, 12 h day/night). All animals received adequate care in compliance with laboratory practice guidelines, and the experimental protocol was approved by the Ethics Committee of The Affiliated Wuxi NO.2 People's Hospital of Nanjing Medical University. To induce an experimental autoimmune myositis (EAM) model, the mice were immunized subcutaneously with 100 μl of 50% complete Freund's adjuvant (Sigma-Aldrich, St. Louis, MS) containing 1.5 mg myosin and 5 mg/ml *Mycobacterium tuberculosis* (BD Biosciences, Franklin Lakes, NY) at the left hind limb and boosted at the tail base and flanks twice weekly as stated in earlier reports (26). The mice were injected intraperitoneally with 500 ng pertussis toxin (Sigma-Aldrich, St. Louis, MS) immediately after each immunization. The control group received saline/complete Freund's adjuvant and pertussis toxin twice. On day 14 after the first immunization, the serum and muscle tissues were collected for further assay.

### PBMC Isolation and *in vitro* Macrophage Differentiation

Human peripheral blood was collected, and peripheral blood mononuclear cells (PBMCs) were isolated via density centrifugation (400 × *g*, 30 min) by human lymphocyte separation medium (Solarbio Life Sciences, China) according to the manufacturer's instruction. The mononuclear cells were washed with phosphate-buffered saline for 5 min at 400 × *g* and 4°C. For macrophage differentiation, PBMCs were cultured with Roswell Park Memorial Institute media and supplemented with glutamax, 20 ng/ml macrophage colony-stimulating factor and 10% fetal bovine serum for 7 days to cause differentiation into macrophages (Gibco Thermo Fischer, Waltham, MA).

### Cell Transfection

Monocyte-derived macrophages were seeded into a six-well plate and transfected with microRNAs (miRNAs) (50 nM), small-interfering RNA (siRNA) (50 pmol), or plasmid (5 μg) using Lipofectamine 3000 (Invitrogen, Carlsbad, CA, USA) at 37°C according to the manufacturer's instruction. After 24 h, the cells underwent further experimentation. MiR-146a mimics, miR-146a inhibitor, and negative control miRNA were obtained from GenePharma (Shanghai, China). The negative control (NC) siRNA, REG3A siRNA, IL-17RA siRNA, pcDNA3.1-NC, and pcDNA3.1-hREG3A were synthesized by Invitrogen (Carlsbad, CA, USA).

### Macrophage Migration Assay

The cells (2 × 10^5^) were suspended in the free serum medium before they were added to the upper chamber of Transwell 96-Well. The medium containing 10% human serum was used as a chemoattractant in the lower chamber. After incubation for 24 h, the invaded cells into the lower chamber were stained with crystal violet. The migrated cells were counted, and photomicrographs were taken under an Olympus inverted microscope (IX71, Olympus, Japan).

### Real-Time Quantitative PCR

Total RNA from muscle tissues and cells was prepared using Trizol reagent (Invitrogen, Carlsbad, CA) according to the manufacturer's instructions. Briefly, complementary DNA (cDNA) was synthesized from 1 μg RNA using SuperScript™ II Reverse Transcriptase (Invitrogen, Carlsbad, CA). Real-time PCR was performed with SYBR Green master Mix (Thermo Fisher Scientific, Waltham, MA) and ABI 7500 sequence detection system (Applied Biosystems, Foster City, CA). Data were analyzed by the 2^(−ΔΔCt)^ method. The primers for miR-146a (Qiagen, MS00001638) and RNU6-2 (Qiagen, MS00033740) were purchased from Qiagen. Each sample was measured in triplicate, and the relative messenger RNA (mRNA) expression was normalized using glyceraldehyde 3-phosphate dehydrogenase/U6. The quantitative PCR thermocycling conditions were as follows: 94°C for 5 min; followed by 40 cycles at 94°C for 30 s, and 60°C for 30 s. The sequences of the primers used for PCR amplification are listed in Table 1.

**Table 1:** The sequences of the primers used for PCR amplification.

**Gene**		**Primer sequence**
hIFN-γ	Sense	GCATCGTTTTGGGTTCTCTTG
	Antisense	AGTTCCATTATCCGCTACATCTG
hIL-17	Sense	AACCTGAACATCCATAACCGG
	Antisense	ACTTTGCCTCCCAGATCAC
hREG3A	Sense	CCTCAAGTCGCAGACACTATG
	Antisense	CTTTGGGACAGCGGATCC
mREG3A	Sense	AAGACATCTGGATTGGGCTC
	Antisense	CACGGTTGACAGTAGAGGAAG
mIL-17	Antisense	TCCAGAATGTGAAGGTCAACC
	Sense	TATCAGGGTCTTCATTGCGG
mIFN-γ	Antisense	TCCAGAATGTGAAGGTCAACC
	Sense	TATCAGGGTCTTCATTGCGG
GAPDH	Sense	GGAGCGAGATCCCTCCAAAAT
	Antisense	GGCTGTTGTCATACTTCTCATGG

*The primers for miR-146a (Qiagen, MS00001638) and RNU6-2 (Qiagen, MS00033740) were purchased from Qiagen*.

### Enzyme-Linked Immunosorbent Assay

Briefly, the whole blood from mice was centrifuged at 8,000 × *g* for 15 min, and serum was collected. The levels of interferon gamma (IFN-γ) and IL-17A were quantified by ELISA commercial kits (R&D system, Minneapolis, MN).

### Western Blot

Cells and muscle tissues were homogenized in RIPA buffer (25 mM Tris–HCl pH 7.6, 150 mM NaCl, 1% NP-40, 1% sodium deoxycholate, 0.1% sodium dodecyl sulfate) containing 1 mmol/L phenylmethylsulfonyl fluoride. After centrifugation for 10 min, the protein concentration in the supernatant was determined using Pierce™ BCA Protein Assay Kit (Thermo Fisher Scientific, Waltham, MA). The protein was separated by 10% sodium dodecyl sulfate polyacrylamide gel electrophoresis and transferred onto a polyvinylidene fluoride membrane. After blocking with 1.5% bovine serum albumin in Tris-buffered saline, the membrane was incubated with indicated primary antibodies and horseradish peroxidase-conjugated secondary antibody. The bands were detected by ECL reagent (Cell Signaling Technology, MA, United States) using Western blot detection system according to the specification of manufacturer's instructions.

### Immunohistochemistry

Muscle tissue were fix in 10% formalin and embedded in paraffin. Slices (5 μm) of muscle tissues were deparaffinized in xylene and subjected to antigen retrieval. The sections were immunostained with primary REG3A antibody (Novus biologicals, #NBP2-24763) at room temperature for 1 h and secondary antibody for 30 min in a humidified chamber. The positive cells were detected by 3,3′-Diaminobenzidine solution according to manufacturer's instruction.

### Statistical Analysis

Data were analyzed using SPSS 17.0 software package (SPSS Inc., IL, USA) and expressed as mean ± SEM. Statistical comparisons between two groups were performed using the Student's *t*-test. A *p* < 0.05 was considered statistically significant.

## Results

### The mRNA Expression Levels of REG3A Increased and miR-146a Decreased in PM/DM Patients

**Figure 1:**
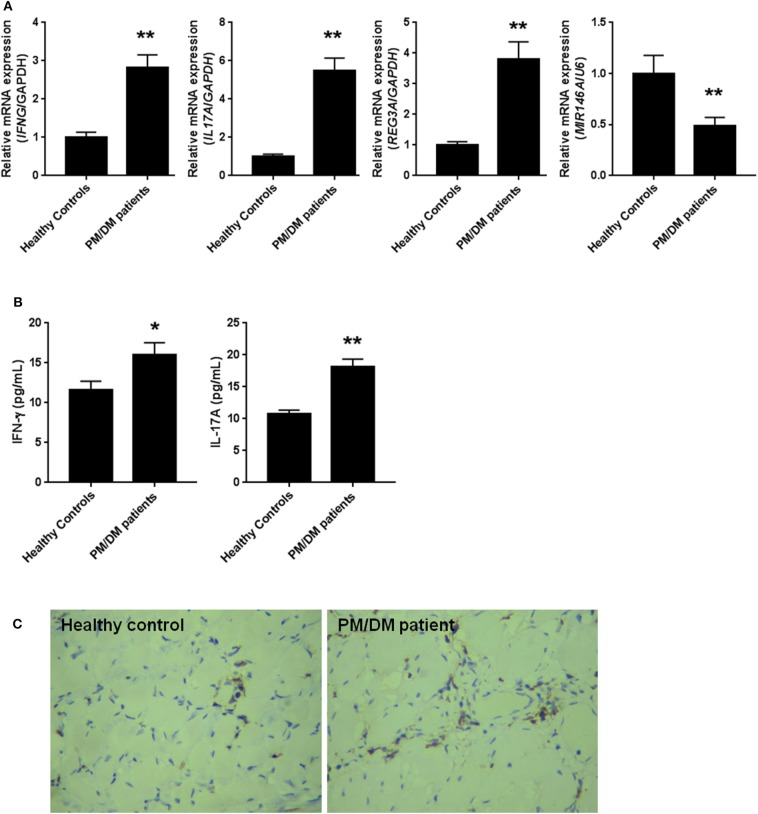
The levels of regenerating islet-derived protein 3-alpha (REG3A) was increased and miR-146a was decreased in polymyositis and dermatomyositis (PM/DM) patients. **(A)** Peripheral blood mononuclear cells (PBMCs) were isolated from patients (*n* = 25) and the healthy controls (*n* = 20) and the messenger RNA (mRNA) levels of interferon gamma (IFN-γ), interleukin (IL)-17A, REG3A, and miRNA-146a were determined by real-time PCR. The relative mRNA expression was normalized using glyceraldehyde 3-phosphate dehydrogenase (GAPDH)/U6. **(B)** The levels of IFN-γ and IL-17A in serum from patients and the healthy controls were determined by ELISA assay. **(C)** The REG3A levels from patients and the healthy controls were determined by immunohistochemistry. Data are shown as means ± SEM. ^*^p < 0.05, ^**^p < 0.01 in comparison with the healthy controls. PBMCs were obtained from 20 healthy controls and 25 patients with PM/DM.

PM/DM are considered as the two most prevalent inflammatory myopathies, which are characterized by the increasing levels of inflammatory cytokines such as IFN-γ and IL-17A. The mRNA expression levels in PBMCs isolated from patients and the healthy controls were detected by real-time PCR. As we expected in Figure 1A, the mRNA expression levels of IFN-γ and IL-17A were remarkably higher in PBMCs from PM/DM patients compared with healthy controls group (*p* < 0.01). Likewise, in comparison with the control group, mRNA expression levels of REG3A was significantly (*p* < 0.01) higher in PBMCs from PM/DM patients, while miR-146a expression was markedly downregulated (*p* < 0.01). Moreover, the levels of IFN-γ and IL-17A in serum from PM/DM patients were significantly increased compared with the healthy controls (Figure 1B). Immunohistochemistry analysis showed that REG3A expression level in the muscle tissue from PM/DM patients was much higher than the healthy controls (Figure 1C).

### The mRNA Expression Levels of REG3A Increased and miR-146a Decreased in an EAM Murine Model

**Figure 2:**
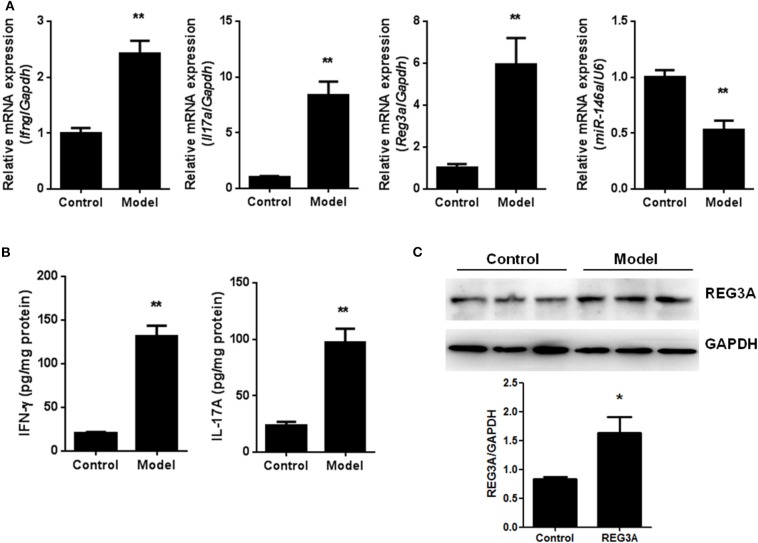
The levels of regenerating islet-derived protein 3-alpha (REG3A) and miR-146a in an experimental autoimmune myositis (EAM) model. **(A)** The messenger RNA (mRNA) expression of interferon gamma (IFN-γ), interleukin (IL)-17A, REG3A, and miRNA-146a in muscle tissues were determined by real-time PCR. The relative mRNA expression was normalized using glyceraldehyde 3-phosphate dehydrogenase (GAPDH)/U6. (*n* = 9) **(B)**. The levels of IFN-γFand IL-17A in serum from the EAM mice were determined by ELISA assay (*n* = 9). **(C)** The protein levels of REG3A in muscle tissues were determined by Western blot (*n* = 3). GAPDH was used as the internal controls for Western blot analysis. Bands were quantified using ImageJ. Data are shown as means ± SEM. ^*^p < 0.05, ^**^p < 0.01 in comparison with the control group.

To confirm the levels of REG3A and miR-146a in PM/DM, an EAM murine model was established, and the mRNA levels of IFN-γ, IL-17A, REG3A, and miR-146a were determined by real-time PCR. The data showed that the mRNA expression of miR-146a was remarkably lower in muscle tissues, while significantly higher mRNA expression of REG3A, IFN-γ, and IL-17A in muscle samples were observed. Furthermore, the IFN-γ and IL-17A in serum were determined by ELISA assay. As shown in Figure 2B, the protein levels of IFN-γ and IL-17A in serum were significantly (*p* < 0.01) elevated in the model group compared with the control cohort, indicating that the model was well-established in mice. Consistently, Western blot analysis showed similar increase in the protein levels of REG3A in muscle tissues compared with the control group (Figure 2C). These results suggest the promotion of inflammatory infiltration in the mice muscle tissues with concomitant elevation of REG3A expression, while reduced levels of miR-146a expression was observed.

### miR-146a and REG3A Regulate Macrophage Migration

**Figure 3:**
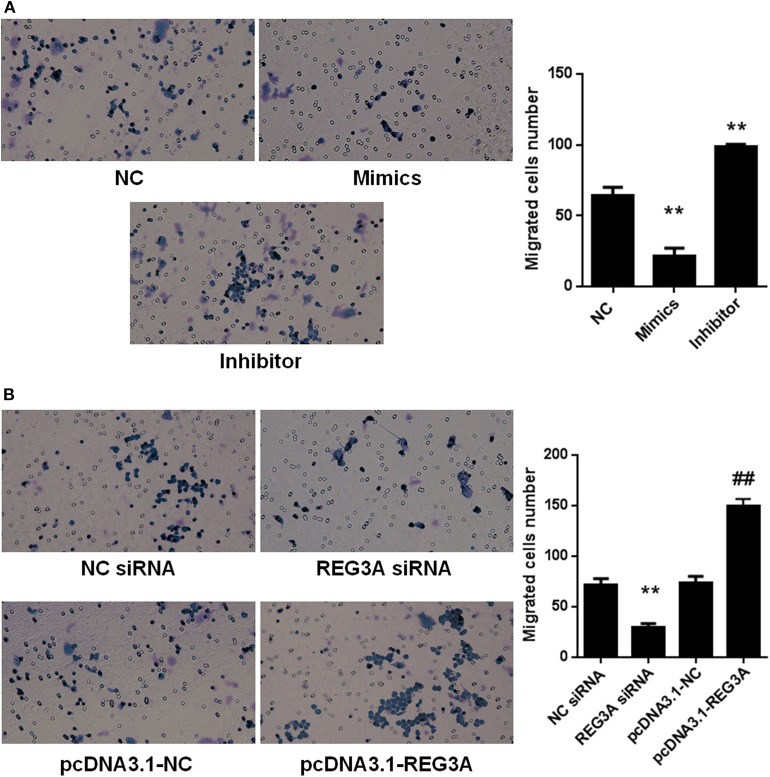
The effects of miR-146a and regenerating islet-derived protein 3-alpha (REG3A) on macrophage migration. **(A)** Monocyte-derived macrophages from the PBMCs of the healthy donors (*n* = 3) were transfected with the negative control (NC) microRNA (miRNA) and miR-146a mimics, miR-146a inhibitors for 24 h. **(B)** Monocyte-derived macrophages from the peripheral blood mononuclear cells (PBMCs) of the healthy donors (*n* = 3) were transfected with the NC siRNA, REG3A siRNA, pcDNA3.1-NC, and pcDNA3.1-REG3A plasmids for 24 h. All treated cells (2 × 10^5^) were suspended and added to the upper chamber of transwell. The medium containing 10% human serum was used as a chemoattractant in the lower chamber. After incubation for 24 h, the invaded cells into the lower chamber were stained with crystal violet. The migrated cells were counted, and photomicrographs were taken under an Olympus inverted microscope (IX71, Olympus, Japan). Data are shown as means ± SEM of three independent experiments. ^*^p < 0.05, ^**^p < 0.01 in comparison with the control group. ^*##*^*p* < 0.05 in comparison with pcDNA3.1-NC group.

Macrophage plays a key role in inflammatory response and the development of PM/DM. Therefore, we aimed to probe the effect of miR-146a and REG3A on macrophage migration. First, monocytes were isolated from the PBMCs of the healthy donors and differentiated into macrophages. Monocyte-derived macrophages of healthy donors were transfected with the NC miRNA, miR-146a mimics, and miR-146a inhibitors for 24 h. As shown in Figure 3A, the numbers of migrated cells as indicated by the transwell assay showed a substantial decrease (*p* < 0.05) in miR-146a mimics group compared with the NC group (Figure 3A). Conversely, a significant increase (*p* < 0.01) in migratory cell numbers was observed upon treatment with miR-146a inhibitors compared with the NC group (Figure 3A). Next, the macrophages were transfected with the NC-siRNA, REG3A-siRNA, pcDNA3.1-NC, and pcDNA3.1-REG3A plasmids for 24 h. As depicted in Figure 3B, the numbers of migrated cells were substantially decreased (*p* < 0.05) after transfection with REG3A-siRNA compared with the NC-siRNA group. Likewise, overexpression of REG3A with pcDNA3.1-REG3A plasmids in macrophage resulted in migratory cell numbers markedly increased (*p* < 0.05) compared with the pcDNA3.1-NC group. Taken together, these findings indicate that miR-146a and REG3A regulated macrophage migration.

### IL-17A Induced REG3A Expression in Macrophage

**Figure 4:**
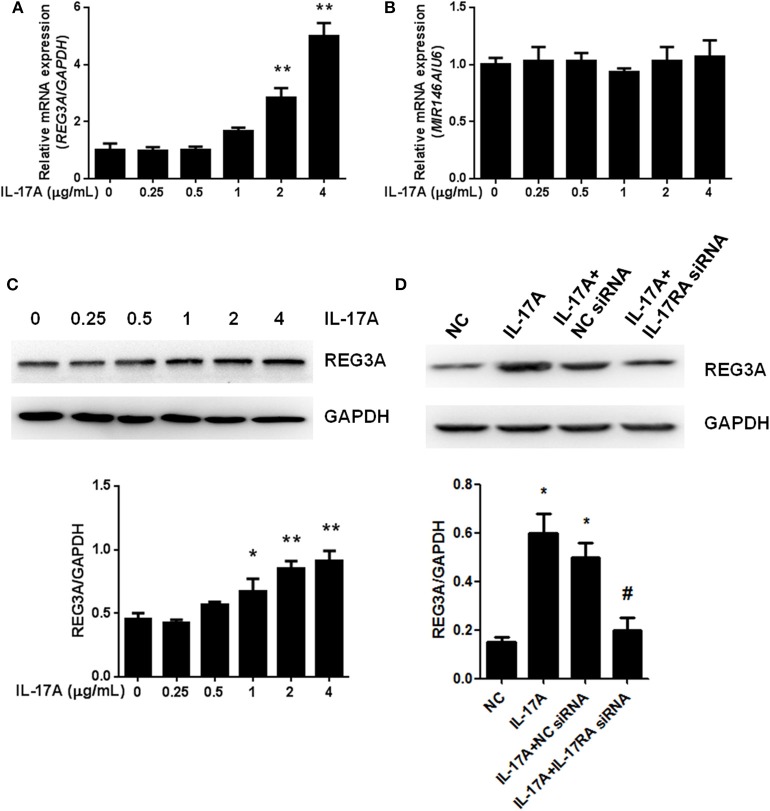
Interleukin (IL)-17A induced regenerating islet-derived protein 3-alpha (REG3A) expression in macrophage. **(A)** Monocyte-derived macrophages from the peripheral blood mononuclear cells (PBMCs) of the healthy donors (*n* = 3) were treated with the different concentrations of IL-17A for 24 h. The messenger RNA (mRNA) expression of REG3A was determined by real-time PCR. Glyceraldehyde 3-phosphate dehydrogenase (GAPDH) was used as the internal controls. **(B)** Monocyte-derived macrophages from the PBMCs of the healthy donors (*n* = 3) were treated with the different concentrations of IL-17A for 24 h. The mRNA expression of miR-146a was determined by real-time PCR. U6 was used as the internal controls. **(C)** Monocyte-derived macrophages from the PBMCs of the healthy donors (*n* = 3) were treated with the different concentrations of IL-17A for 24 h. The protein levels of REG3A were determined by Western blot. GAPDH was used as the internal controls. **(D)** Monocyte-derived macrophages were transfected with the NC small-interfering RNA (siRNA) and IL-17RA siRNA in the absence or presence of IL-17A for 24 h. The protein levels of REG3A were determined by Western blot. GAPDH was used as the internal controls. Western blot bands were quantified using ImageJ and normalized to GAPDH. Data are shown as means ± SEM of three independent experiment. ^*^p < 0.05, ^**^p < 0.01 in comparison with the control group. ^#^*p* < 0.05 in comparison with IL-17A + NC siRNA group.

IL-17-mediated inflammation is crucial for autoimmune diseases, which induce a set of gene expression through IL-17 receptor pathway. The relationship among IL-17A miR-146a and REG3A in macrophage remains unclear especially among PM/DM patients. Monocyte-derived macrophages obtained from healthy donors were treated with different concentrations of IL-17A for 24 h. Notably, we found that the mRNA expression of REG3A significantly (*p* < 0.01) increased by IL-17A treatment in a dose-dependent manner (Figure 4A). Conversely, similar treatment with diverse concentrations of IL-17A did not affect mRNA expression of miR-146a (Figure 4B). Next, Western blot analysis confirmed the induction of REG3A expression by IL-17A in a dose-dependent manner (Figure 4C). Finally, we tested whether IL-17 induced REG3A expression through IL-17RA receptor. Western blot analysis showed that the IL-17A treatment significantly (*p* < 0.05) increased the expression of REG3A compared with the NC group. Treatment with IL-17A + NC siRNA significantly induced REG3A expression, while the inhibition of REG3A expression was observed upon treatment with IL-17A + IL-17RA siRNA (Figure 4D). Altogether, these results show that IL-17A can induce the expression of REG3A in macrophage through IL-17RA pathway.

### miR-146a Regulates REG3A Expression in Macrophage

**Figure 5:**
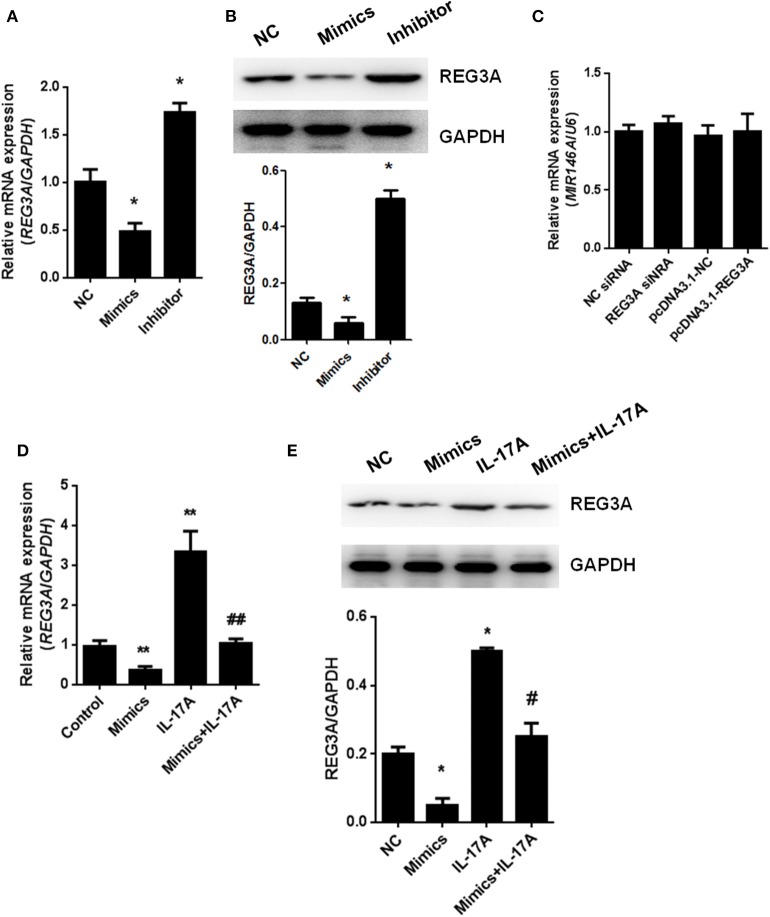
miR-146a regulates regenerating islet-derived protein 3-alpha (REG3A) expression in macrophage. **(A,B)** Monocyte-derived macrophages from the peripheral blood mononuclear cells (PBMCs) of the healthy donors (*n* = 3) were transfected with negative control (NC) microRNA (miRNA), miR-146a mimics, or miR-146a inhibitor for 24 h. The messenger RNA (mRNA) and protein levels of REG3A were determined by real-time PCR and Western blot. Glyceraldehyde 3-phosphate dehydrogenase (GAPDH) was used as the internal controls. **(C)** The cells were transfected with NC small-interfering RNA (siRNA), REG3A siRNA, pcDNA3.1-NC, and pcDNA3.1-REG3A plasmids for 24 h. The mRNA levels of miR-146a were determined by real-time PCR. U6 was used as the internal controls. **(D,E)** The cells were transfected with NC miRNA or miR-146a mimics in the absence or presence of IL-17A for 24 h. The mRNA and protein levels of REG3A were determined by real-time PCR and Western blot. GAPDH was used as the internal controls. Data are shown as means ± SEM of three independent experiments. ^*^p < 0.05, ^**^p < 0.01 in comparison with the control group. ^#^*p* < 0.05, ^##^*p* < 0.01 in comparison with IL-17A treatment group.

To understand the relationship between miR-146a and REG3A, the regulation of the expression of the latter by the former was established in monocyte-derived macrophages via transfection with NC miRNA, miR-146a mimics, or miR-146a inhibitor for 24 h. Consequently, the real-time PCR and Western blot results showed a significant (*p* < 0.05) increase in expression of REG3A in miR-146a inhibitor-treated group compared with the NC group. Conversely, transfection with miR-146a mimics resulted in the substantial (*p* < 0.05) downregulation of REG3A expression in relation to the NC group (Figures 5A,B). On the other hand, transfection with NC siRNA, REG3A siRNA, pcDNA3.1-NC, and pcDNA3.1-REG3A plasmids for 24 h could not alter the level of miR146a (Figure 5C). Notably, treatment of monocyte-derived macrophages with miR-146a mimics in the absence of IL-17A showed decreased mRNA and protein expression of REG3A in comparison with IL-17A treatment group (Figures 5D,E). These findings suggest that the increased expression of miR-146a might lead to the inhibition of REG3A expression irrespective of the presence of IL-17A.

### miR-146a Inhibits Macrophage Migration via Suppression of REG3A Expression

**Figure 6:**
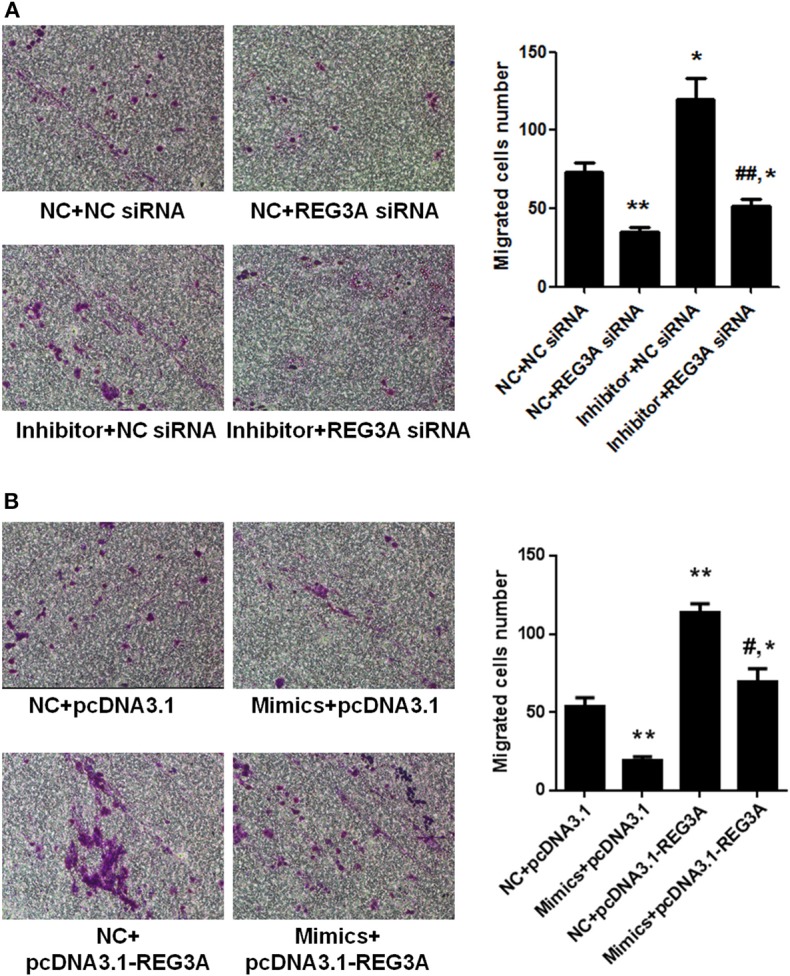
miR-146a inhibited macrophage migration through suppression of regenerating islet-derived protein 3-alpha (REG3A) expression. **(A)** Monocyte-derived macrophages from the PBMCs of the healthy donors (*n* = 3) were transfected with the NC siRNA or REG3A siRNA in the absence or presence of miR-146a inhibitor for 24 h. **(B)** Monocyte-derived macrophages were transfected with the pcDNA3.1-NC or pcDNA3.1-REG3A plasmids in the absence or presence of miR-146a mimics for 24 h. All treated cells (2 × 10^5^) were suspended and added to the upper chamber of transwell well. The medium containing 10% human serum was used as a chemoattractant in the lower chamber. After incubation for 24 h, the invaded cells into the lower chamber were stained with crystal violet. The migrated cells were counted, and photomicrographs were taken under an Olympus inverted microscope (IX71, Olympus, Japan). Data are shown as means ± SEM of three independent experiment. ^*^p < 0.05, ^**^p < 0.01 in comparison with NC + NC siRNA or NC + pcDNA3.1 group. ^#^*p* < 0.05, ^##^*p* < 0.01 in comparison with inhibitor + NC siRNA or NC + pcDNA3.1-REG3A group.

To investigate whether suppression of REG3A by miR-146a has any effect on macrophage migration, we transfected monocyte-derived macrophages obtained from healthy donors with NC siRNA or REG3A siRNA in the absence or presence of miR-146a inhibitor for 24 h. Transwell assay showed that the number of migrated cells significantly decreased (*p* < 0.01) after transfection with NC + REG3A siRNA compared with the NC + NC siRNA group. Contrarily, migratory cell numbers obviously increased upon treatment with inhibitor + NC siRNA (*p* < 0.05). However, treatment with inhibitor + REG3A siRNA resulted in the substantial decrease in migrated cell number compared with the inhibitor + NC siRNA and NC + NC siRNA group (Figure 6A), indicating that inhibition of miR-146a could not promote REG3A-silenced macrophage migration. In addition, we transfected monocyte-derived macrophages with the pcDNA3.1-NC or pcDNA3.1-REG3A plasmids in the absence or presence of miR-146a mimics for 24 h. As shown in Figure 6B, a substantial (*p* < 0.01) decreased number of migrated cells was observed in mimics + pcDNA3.1 group compared with the NC + pcDNA3.1 group. Consistent with results in Figure 3B, transfection of the macrophages with NC + pcDNA3.1-REG3A plasmids led to a significant increase (*p* < 0.05) in migrated cell numbers compared with the NC + pcDNA3.1 group (Figure 6B). However, treatment of the macrophage with mimics + pcDNA3.1-REG3A displayed a significant decrease in cell migration compared with the NC + pcDNA3.1-REG3A group, indicating that the overexpression of REG3A-induced macrophage migration was inhibited by miR-146a treatment. These data suggest that miR-146a may inhibit macrophage migration via suppression of REG3A expression.

## Discussion

This study was designed to test the possibility that miR-146a inhibits macrophage migration by downregulating REG3A expression, which might contribute to the development of PM/DM. The following observations emerged from the present study: the findings support the evidence that the mRNA expression levels are increased in IFN-γ, IL-17A, and REG3A, while miR-146a expression is reduced in PBMCs from PM/DM patients. An EAM murine model increased mRNA expression of REG3A as well as decreased miR-146a mRNA expression. miR-146a inhibited macrophage migration, while REG3A promoted PBMC-differentiated macrophage migration. IL-17A induced REG3A expression in macrophage in a dose-dependent manner *in vitro*. while miR146a inhibited expression of REG3A in macrophage. Notably, inhibition of macrophage migration by miR-146a was via the reduction in REG3A expression.

Most PM/DM patients experience muscle weakness, skin lesions, fatigue, and breathing difficulties, which majorly affect the quality of their daily life. An earlier study has observed lack of evidence-based theory to identify appropriate treatment options for PM/DM owing to unclear pathology underlying these conditions (7). However, as an idiopathic inflammatory myositis, it has been postulated that inflammation is the primarily underlying mechanism of the aforementioned symptoms of these diseases (27). Over the years, physician experience and empirical data have played key roles in selecting treatments for PM/DM, but difficulty in timely diagnosing PM/DM caused by similar symptoms of the disease to those of myositis has hampered this practice (28). Clinically, previous review showed that corticosteroids yielded only a modest benefit, while azathioprine and methotrexate were demonstrated to be effective in only 8 out of 35 trials (25, 26, 29, 30). Thus, understanding the detailed mechanism underlying the signs and symptoms of PM/DM will aid in the identification of pharmacological biomarkers and the development of effective therapeutic approaches for the cure of these diseases.

Emerging evidence suggests that miRNAs, particularly miR-146a, is reported to play a vital role in the pathological process of inflammatory diseases like PM/DM (31). Likewise, the activation of downstream IL-17A and IFN-γ signaling pathways has been observed to promote inflammation in several diseases (28, 32). Besides, it has been reported elsewhere that the antimicrobial protein REG3A expressed by keratinocytes is induced by IL-17 via activation of keratinocyte-encoded IL-17RA (23). These emerging pieces of evidence, therefore, support the concept that IL-17A, miR-146a, and REG3A are involved in the pathogenesis of inflammatory disease such as PM/DM. In the present study, it was shown that IL-17A and IFN-γ was highly expressed in PBMCs from PM/DM patients with higher mRNA expression of REG3A and lower miR-146a expression (Figure 1). This result corroborates earlier findings that higher levels of IL-17 and its proinflammatory counterparts IL-17RA are produced in PM/DM patients by T helper cells (33, 34). Consistently, the *in vivo* data obtained from mice model showed that autoimmune myositis resulted in increased mRNA expression of REG3A as well as decreased miR-146a expression (Figure 2). Thus, the associative effect of these biological molecules is relevant for the immune response-inflammatory processes in the PM/&DM pathophysiology. Inflammation is a necessary defensive reaction to several physiological conditions involving immune cells, blood vessels, and molecular mediators. Nevertheless, it contributes significantly to the pathogenesis of various diseases, including PM/DM (1). The primary pathological process in these disorders occurs with inflammatory infiltrates in the muscles (5). Notably, macrophage-mediated inflammatory infiltrates are critical for a variety of human inflammatory muscle disorders as macrophages are key cell types involved in orchestration and modulation of the repair process (31, 35). In addition, unresolved inflammatory infiltration facilitated by macrophage may lead to persistent muscle tissue destruction by immune cells or collagen deposition (36). Therefore, therapeutic strategies targeting inflammatory response in PM/DM may be a promising approach to manage these conditions. A previous report has established the regulation of inflammation infiltration in PM/DM by macrophages via targeting tumor necrosis factor receptor-associated factor 6 and affecting IL-17/intercellular adhesion molecule 1 pathway (11). However, the mechanism underlying inflammatory macrophage infiltration in muscle tissue restoration among PM/DM patients is incompletely understood. Earlier studies have shown that IL-17 has strong influence on the pathogenesis of several other autoimmune diseases, such as rheumatoid arthritis, multiple sclerosis, and psoriasis (37–39). In addition, in muscle tissue, IL-17 coupled with other proinflammatory cytokines, such as IFN-γ produced by monocytes and innate immune responses, acts to potentiate immune responses, which might lead to destruction of muscle tissues (40). Consequently, the increased expression of REG3A induced by IL-17A (Figure 4) might exacerbate the PM/DM condition, thereby potentially heightening immune responses. On the other hand, REG3A is established to be highly expressed in skin cells in concert with IL-17 during psoriasis and wound healing (23), which might play a critical role in muscle tissue healing in PM/DM. Thus, future researches can explore the development of IL-17A/REG3A targeted antibody therapies and their prospect in treating PM/DM. Besides, since REG3A is reported to promote wound healing in skin injuries (41), its role in improving muscular cell growth and tissue restoration in PM/DM patients will be given the needed attention in our subsequent investigations.

Actually, miR-146a expression levels were observed to be markedly lower in either patients and mice models than healthy subjects or the control group, respectively (Figures 1, 2). In addition, experimental treatment with mimics or inhibitors of miR-146a impacted on REG3A expression levels as well as macrophage migratory capacity (Figures 3, 5, 6). Based on these and other findings, we hypothesized that miR-146a and IL-17A regulate the expression of REG3A in PM/DM patients. Although earlier report has recognized the significance of miR-146a in PM/DM (38, 42), its relationship with REG3A has not been established yet. On the account of a previous investigation, which has suggested the abnormal expression of miR-146a and its negative regulatory role in inflammation (43), this study unearths possible mechanism underlying macrophage-mediated inflammatory infiltrates via REG3A (Figure 3). Specifically, increased miR-146a expression was observed to inhibit REG3A expression (Figure 5). Suppression of REG3A expression by miR-146a resulted in the inhibition of macrophage migratory capacity (Figure 6). In the present report, IL-17A and miR-146a inhibitors had positive influence on REG3A expression coupled with macrophage migration, which were counteracted via transfection with REG3A siRNA. Altogether, these findings suggest the relationship between miR-146a and REG3A as well as their involvement in PM/DM pathophysiology. MiR-146a is well established to inhibit cell migration and mediate suppression of inflammatory response in human adipocytes (44, 45). Conversely, REGs are reported to be highly expressed in diseases such as hepatic injury and inflammatory bowel disease-related colonic inflammation (22, 46). Preliminary, this report has evidenced that miR-146a and IL-17A can regulate the pathogenesis of PM/DM via targeting of REG3A, which, to the best of our knowledge, has not been established in other reports. However, the interplay between miR-146a, REG3A, and its related pathways such as defensins and innate immune systems in the pathogenesis of PM/DM will be investigated comprehensively in future studies.

In summary, the present study for the first time proves that reduced miR-146a in PM/DM promotes REG3A expression and inflammatory macrophage migration. We believe that this preliminary evidence will contribute to the understanding of the mechanism underlying PM/DM pathogenesis, which can possibly facilitate diagnosis of the disease. Nonetheless, future investigations are needed to further elucidate other REGs and targeting proteins involve in PM/DM pathogenesis to develop appropriate treatment approaches.

## Data Availability Statement

The raw data supporting the conclusions of this article will be made available by the authors, without undue reservation, to any qualified researcher.

## Ethics Statement

The experimental protocol was approved by the Ethics Committee of The Affiliated Wuxi No. 2 People's Hospital of Nanjing Medical University. All patients provided written informed consent for the use of their tissues and data prior to the study. All animals received adequate care in compliance with laboratory practice guidelines and the experimental protocol was approved by the Ethics Committee of The Affiliated Wuxi No. 2 People's Hospital of Nanjing Medical University.

## Author Contributions

All authors listed have made a substantial, direct and intellectual contribution to the work, and approved it for publication.

### Conflict of Interest

The authors declare that the research was conducted in the absence of any commercial or financial relationships that could be construed as a potential conflict of interest.
